# The Kinetics of Anti-HLA Antibodies in the First Year after Kidney Transplantation: In Whom and When Should They Be Monitored?

**DOI:** 10.1155/2018/8316860

**Published:** 2018-04-23

**Authors:** Maria Cristina Ribeiro de Castro, Erick A. Barbosa, Renata P. Souza, Fabiana Agena, Patrícia S. de Souza, Gabriella Maciel, Hélcio Rodrigues, Nicolas Panajotopoulos, Daísa S. David, Flávio J. de Paula, Elias David-Neto

**Affiliations:** ^1^Renal Transplantation Service, Hospital das Clínicas, University of São Paulo School of Medicine, São Paulo, SP, Brazil; ^2^Laboratory of Immunology (LIM 19), Heart Institute, University of São Paulo School of Medicine, São Paulo, SP, Brazil

## Abstract

The impact of the kinetics of the anti-HLA antibodies after KTx on the occurrence of acute rejection as well as the better time-point to monitor anti-HLA Abs after transplantation is not completely defined. This prospective study followed 150 patients over 12 months after transplantation. Serum IgG anti-HLA Abs were detected by single antigen beads after typing donors and recipients for loci A, B, C, DR, and DQ. Before KTx, 89 patients did not present anti-HLA Abs and 2% developed “de novo” Abs during the 1st year, 39 patients were sensitized without DSAs, and 13% developed DSA after surgery; all of them presented ABMR. Sensitized patients presented higher acute rejection rates (36.4% versus 13.5%, *p* < 0.001), although 60% of the patients did not present ABMR. Patients, in whom DSA-MFI decreased during the first two weeks after surgery, did not develop ABMR. Those who sustained their levels presented a rate of 22% of ABMR. 85% of patients developed ABMR when MFIs increased early after transplantation (which occurred in 30% of the DSA positive patients). In the ABMR group, we observed an iDSA-MFI sharp drop on the fourth day and then an increase between the 7th and 14th POD, which suggests DSA should be monitored at this moment in sensitized patients for better ABMR prediction.

## 1. Introduction

Anti-HLA Abs (anti-HLA Abs) as well as donor-specific alloantibody (DSA) is an increasingly common finding in renal transplant candidates [1, 2]. Sensitization to human leukocyte antigens (HLA) occurs mainly through pregnancies, blood transfusions, and transplantation. Anti-HLA sensitized patients have a high incidence of antibody-mediated rejection (ABMR) in the first few weeks after transplantation [3, 4].

The importance of HLA matching and the presence of pretransplant anti-HLA antibodies, on the outcome of renal transplantation, have been studied [5, 6]. However, the clinical relevance of the dynamics of preformed anti-HLA antibody after transplantation has not been well described.

In a large multicenter study, Terasaki and Ozawa found that the prevalence of anti-HLA Abs after kidney transplantation, in the long-term, was 20.9% and those patients who developed anti-HLA antibodies had lower graft survival, suggesting that the appearance of circulating antibodies precedes rejection episodes [1].

We have previously studied the kinetics of anti-HLA Abs after kidney transplantation using ELISA-Panel Reactive Antibodies (ELISA-PRA) determination and showed that the increase in ELISA-PRA levels was associated with the occurrence of acute antibody-mediated rejection [7]. Also, in a retrospective analysis of anti-HLA Abs after KTx, we have observed that most of the patients with pre-Tx DSA, whose graft survived after 6 years of follow-up, had cleared/decreased their pre-Tx Abs after KTx [8].

In this study, we have prospectively evaluated the kinetics of the anti-HLA Abs antibodies and DSAs after kidney transplantation and its impact on the occurrence and severity of acute rejection episodes. We have also tried to identify the best time-point to monitor anti-HLA Abs in the first year after kidney transplantation.

## 2. Methods

### 2.1. Patients

This is a prospective and observational study that evaluated 1350 sera of 150 adult patients (≥18 years) who were submitted to a non-HLA identical, isolated kidney transplant. Patients were followed over a period of 12 months after transplantation or until graft loss or death. All participants signed informed consent approved by the Institutional Committee of Ethics in Research (# 0233/11).

All patients (*n* = 223) who received a kidney transplant at our center between July 2011 and June 2012 were invited to participate. Out of them, 53 were not included due to (a) younger age than 18 y (*n* = 16); (b) declining to participate (*n* = 27); (c) multiple organ transplants (*n* = 10). Twenty patients were excluded after transplantation: 6 died and 6 lost their grafts very early after transplantation (none due to ABMR) and 8 were lost from follow-up. Therefore, 150 patients were enrolled in this study.

### 2.2. HLA Typing

All donors and recipients were HLA A, B, C, DRB1, and DQB1 typed by polymerase chain reaction single strand polymorphisms (PCR-SSP) or polymerase chain reaction sequence specific oligonucleotides (PCR-SSO, One Lambda, Canoga Park, CA).

### 2.3. Pretransplant Cross-Match

Pretransplant DSA and inacceptable mismatches were not used to stratify transplant risk. At the time of the transplant, all patients had a negative pretransplant AGH-CDC T-cell cross-match (XM) as well as long-incubation B cell XM. The presence of IgM antibodies was excluded by testing in the presence of Dithiothreitol‎ (DTT). Sensitized patients who received a live donor kidney were also submitted to T and B flow-cytometry cross-match (FCXM) and cleared to transplant if negative: after isolating peripheral T and B lymphocytes, viability was checked, and the concentration was adjusted to 2.5–3.5 × 10^6^ per mL. The donor cells were incubated with recipient serum at 4°C and then washed in 5% fetal calf serum in phosphate-buffered saline. Fluorescein isothiocyanate conjugated with anti-human globulin was added, followed by a 2-min incubation. Fluorochromes conjugated with monoclonal antibodies specific for T cells (PerCP anti-CD3) and B cells (PE anti-CD19) were added, followed by 30-min incubation at 4°C and a wash step. The crossmatches were acquired by the flow cytometer, a BD FACS Calibur fluorescence-activated cell sorter using a 1024-channel log scale (Becton-Dickinson Biosciences, San Jose, CA).

### 2.4. Panel Reactive Antibodies (PRA)

Blood samples were collected before transplantation (and before the administration of any induction therapy) as well as on days 4, 7, 14, 30, 90, 180, and 360 after the surgery. All pre- and posttransplant sera were screened by Lab Screen Mixed (One Lambda, Canoga Park, CA) to determine the presence of class I and class II anti-HLA Abs of the IgG isotype. Blood samples were analyzed prospectively. Raw MFI data was analyzed

The cPRA was computed using frequencies found in the donor population in our area. Positive samples were subsequently analyzed to determine antibody specificity using by solid-phase assay single HLA-coated microspheres (Luminex® - One Lambda, Canoga Park) in all patients with a PRA higher than 0%. According to our laboratory standardization, MFI cut-off value of 1500 was considered positive and negative MFI values below this cut-off point of 1500, either for class I and II antigens, and for preexistent and for “de novo” antibodies. We used the immune-dominant DSA (iDSA), that is, the anti-donor HLA antibody with the highest mean fluorescent intensity (MFI) for statistics when more than one antibody was detected. For this study, we defined change in posttransplant DSA as a 20% increase or decrease on MFI, considering that all specimens were analyzed by the same laboratory, using the same technique with a sensitive method from the same manufacture and considering that in our laboratory chances are lower than 10% in 95% of the tests. In our laboratory, only variations greater than 10% on DSA levels are considered significant. All patients who presented a posttransplant “de novo” antibody had their pretransplant sera retested by single antigen beads, to confirm that these antibodies were not already present immediately before transplantation. EDTA was used to ensure that a prozone effect was not present.

### 2.5. Immunosuppression

All patients received induction therapy: 108 received Basiliximab (Novartis Biosciences, Basel, Switzerland), and 42 received Thymoglobulin (Genzyme Corporation, Cambridge, USA). Depleting antibodies were indicated if there was immunological risk (PRA higher than 10% or DSA presence) or a long (higher than 24 hours) cold ischemia time. Maintenance immunosuppression consisted of Tacrolimus, Sodium Mycophenolate, and Prednisone. There were no differences in the long-term immunosuppressive policy concerning the type of drugs or their doses in sensitized and no sensitized patients. TCMR classified as Banff I were treated with Methylprednisolone pulses (500 mg per 3 days). TCMR classified as Banff II-III were treated with Thymoglobulin 6 mg/Kg over 4–8 days (Sanofi-Aventis). ABMR was treated with plasmapheresis (6 sessions), and a course of IVIg (2 g/kg) and Rituximab 500–1000 mg (Mabthera from Roche Pharmaceuticals) after apheresis.

### 2.6. Allograft Rejection

Rejections were biopsy-proven in all cases and classified using Banff 2009 criteria [9]. C4d staining was performed by indirect immunofluorescence technique, using monoclonal anti-C4d antibody from Biogenesis (Sandown, NH, MO, EUA) and considered positive when more than 10% of the peritubular capillaries stained positive. Antibody-mediated rejection (ABMR) was defined when there was (1) histological evidence of tissue injury, (2) C4d positivity, and (3) the detection of circulating anti-HLA donor-specific antibodies. Sensitized patients were always submitted to a protocol biopsy between the 7th and the 10th POD. Biopsies were performed in three situations: between the 7th and 10th POD in patients with cPRA higher than 0 or evolving with delayed graft function and anytime when rejection was suspected. Total number of biopsies performed during the study was 138.

### 2.7. Statistical Analysis

Statistical analysis was performed using the SPSS for Windows, version 21 (SPSS Inc., Chicago, USA). For comparison of means and frequencies of normally distributed variables* T*-tests and Fisher Exact Test were applied. Logistic regression analysis was used to compare the risk of an outcome for a given DSA. A “*p*” value of less than 0.05 was considered as significant.

## 3. Results

The 150 enrolled patients were grouped according to both: the sensitization and the presence/absence of pretransplant DSA.

  Group A: nonsensitized patients (*n* = 89, 59%).
  Group B: sensitized patients with no DSA (*n* = 39, 26%).
  Group C: sensitized patients with DSA (*n* = 22, 15%).

Table 1 describes the demographics of these patients. Groups were similar in terms of recipient and donor age, dialysis before transplantation, donor type, history of transfusions, and number of HLA-DR mismatches.

In group C, there were a higher percentage of women, retransplants, pregnancies, and more Thymoglobulin as induction therapy. Also, group C presented a higher number of HLA A, B, C, and DQ mismatches. Class I PRA was higher in group C than in B (35% versus 6%). In addition, class II PRA was also higher in group C than in B (34% versus 12%).

No statistical difference was observed on the percentage of living and deceased donor transplants in the three groups. To avoid false interpretations due to small number of subjects in each group, living and deceased transplants were analyzed together.

Pretransplant T and B cell FCXM were performed only in the 7 patients who received kidney transplants from living donors out of the 22 patients in group C. In these 7 patients, pretransplant T and B cell FCXM were negative, which allowed us to clear the transplant. T and B cell FCXM was not performed to clear deceased donor transplantation in these DSA positive patients.

### 3.1. Acute Rejection

Table 2 shows the clinical outcomes according to pre-Tx PRA. The overall rate of acute rejection in this study was 20% (30 episodes in 150 patients). In 12 patients (40%), the rejection was characterized as active antibody-mediated (all C4d positive) and in 18 patients (60%) as cell mediated rejection. Acute cellular rejection occurred on a median day 85 and ABMR on a median day 12 after transplant. The incidence of acute rejection was higher in group C (36.4%) than in groups A and B (13.5% and 25.6%, respectively, *p* =< 0.001). The percentage of ABMR was also higher in group C than in groups B and A (100% versus 40% versus 0%, *p* =< 0.0001). No difference at one-year patient and graft survival was detected among groups. No biopsy showed features of chronic antibody-mediated rejection.

The presence of pretransplant DSA showed an odds ratio of 2.7 for acute rejection and 17.7 for ABMR. Patients without ABMR had better 1-year eGFR as compared with patients with ABMR (54 ± 20 versus 42 ± 9 ml/min/1.73 m^2^, *p* = 0.002, respectively), There were no differences in the urinary protein-to-creatinine ratio at one year (0.24 ± 0.39 versus 0.81 ± 1.28, *p* = 0.1715).

### 3.2. Kinetics of HLA Antibodies after Transplantation

*Group A (Pretransplant, PRA = 0)*. Out of the 89 nonsensitized patients, 79 (89%) never developed anti-HLA Abs up to one year after KTx, 8 (9%) develop non-DSA anti-HLA Abs, and 2 (2%) developed “de novo” DSA (one anti-HLA class I, detected at day 180, and one anti-HLA class II, detected at day 360 after transplantation). None of these patients in group A developed clinical ABMR.

*Group B (Pretransplant, PRA > 0 no DSA)*. The 39 patients in this group had anti-HLA Abs, but no DSA. One year after KTx, 34 patients (87%) neither changed Classes I or II PRA nor developed “de novo” DSA. Five patients (13%) developed “de novo” DSA, 4/5 (80%), in the first week after transplantation (2 Class I and 2 Class II) and all these 4 patients developed ABMR, in the first month. In the other patient “de novo” anti-HLA class II DSA was detected on the 180th postoperative day and this patient did not develop clinical rejection.

*Group C (PRA > 0 with DSA)*. This group included 22 patients transplanted with detectable pretransplant DSA, but with negative pretransplant T and B AGH-CDC-XM. After transplantation, different DSA kinetics were identified:

- Six patients (27%) decreased by 47% the iDSA-MFI (7329 ± 3932 to 3821 ± 3901, *p* = 0,002) from day 0 to day 14. From day 30 to day 90 they further decreased iDSA-MFI levels by 29% (3206 ± 3258 to 2162 ± 2785, *p* = 0.003). In none of these patients ABMR occurred.
- Nine patients (34%) did not reduce the iDSA-MFI significantly between day 0 and day 14 (4999 ± 5595 versus 3887 ± 4884, *p* = 0.07) and two of them (22%) developed clinical ABMR during the first two weeks.
- Seven patients (32%) had a significant increase in the iDSA-MFI from day 0 to day 14 (6095 ± 2405 versus 10740 ± 4253, *p* = 0.049) and all them (100%) developed ABMR over this period. One graft loss due to ABMR occurred. In these 7 patients, after treatment with plasmapheresis, IVIg, and Rituximab, 6 presented a significant decrease in iDSA-MFI at day 30 (2162 ± 2785, *p* = 0.003) and day 90 (1923 ± 2641, *p* = 0.003). Among them, and between 90 and 360 days, 3 patients eliminated their DSAs.

**Figure 1:**
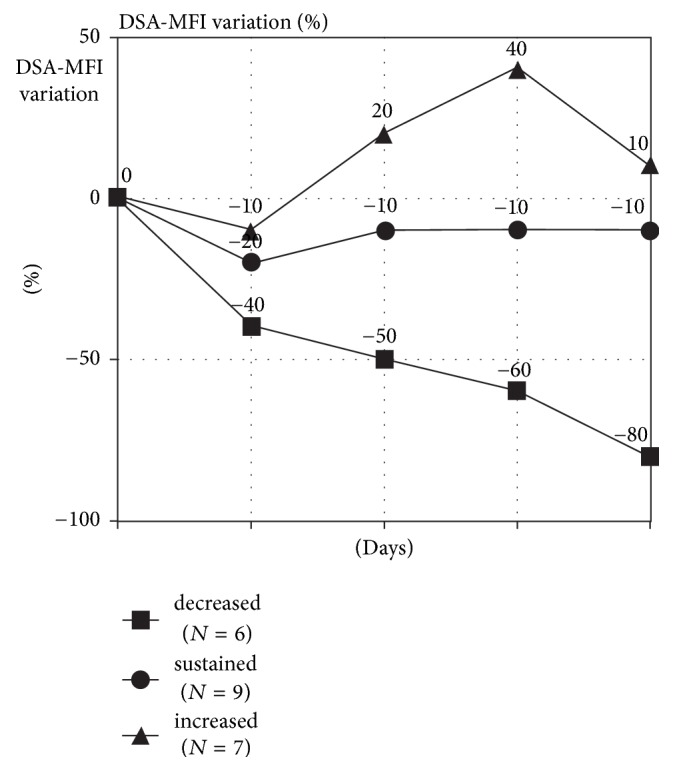
DSA-MFI percentage variation in DSA positive patients over the 1st month.

**Figure 2:**
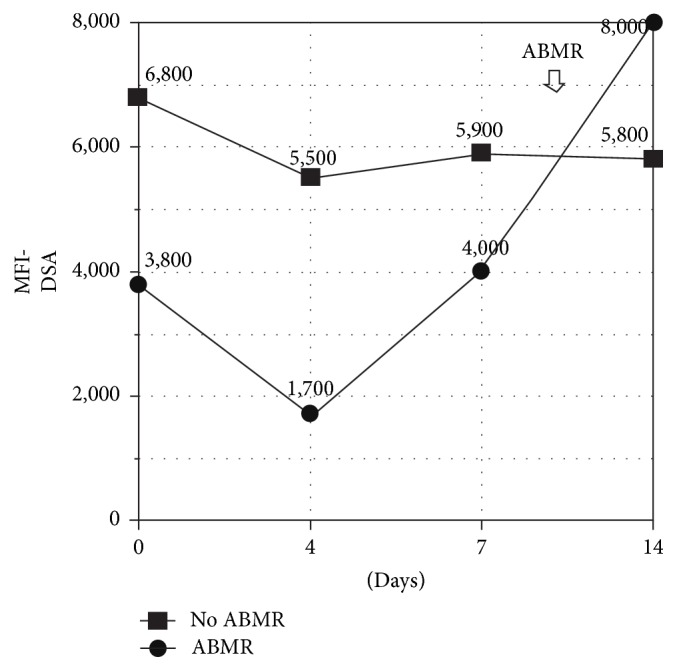
Mean iDSA-MFI in patients with and without ABMR over two weeks.

Not only the kinetics but also the amount of the increase on iDSA-MFI was different among the patients between the 4th and the 7th POD. Patients that presented ABMR, had a 44% mean increase on iDSA-MFI levels, whereas patients without ABMR had only a nonsignificant 8% increase (Figure 2). MFI variation of these groups is shown in Figure 1. No statistical differences were found on 1-year graft survival among all groups (NS).

We could identify risk factors for the appearance of “de novo” DSA or iDSA-MFI increase after surgery. Women were more prone to DSA increase/appearance (19.4 versus 1.2%, *p* = 0.0001, OR = 19.7), as well as retransplants (9.6 versus 6.7%, *p* < 0.001, OR = 1.5), previous pregnancies (23.5 versus 6.3%, *p* = 0.16, OR = 4.6), deceased donor (12.1 versus 3.9%, *p* = 0.14, OR = 3.4), and previous transfusions (13.9 versus 5.1%, *p* = 0.09, OR = 3).

## 4. Discussion

In this study, we observed that only 2% of nonsensitized patients develop “de novo” DSA in the first year and none of them developed ABMR. We have also shown that sensitized patients either with pre- or “de novo” posttransplant DSA have a high risk of ABMR if the DSA-MFI increases in the first week after transplantation what suggests that the 7th day is a good time-point for DSA monitoring.

International consensus on monitoring anti-HLA Abs suggests that nonsensitized patients should be tested at least once between the third and the 12th month after transplantation [10]. Our results led us to conclude that monitoring anti-HLA antibodies, in the first year, in nonsensitized patients may strongly increase costs with a small benefit. On the other side, sensitized patients without DSA at the transplant must be followed with caution, since, in our cohort, 13% of them developed DSA, usually very early after transplantation, and 80% of them developed ABMR. This data changed our policy with DSA search and protocol renal biopsies between the 7th and the 14th POD to detect and treat developing ABMR in sensitized patients without pre-Tx DSA.

In this analysis, among sensitized patients, those transplanted with DSA had a high risk of ABMR. These data were similar with data described by Lefaucheur et al. [2]. In their study, the main factor predicting ABMR was the presence of pretransplant DSA with a ninefold relative risk. In our study, we had a 17.7-fold risk of ABMR. Nevertheless, neither the presence of DSA nor the MFI value could predict ABMR, since 64% of these patients did not course with ABMR.

In this study, the intensity of pre-Tx DSA fluorescence was not associated with the development of ABMR.

Some studies have shown no differences in mean pretransplant MFIs among patients with and without ABMR, although patients with higher levels present higher ABMR [11, 12]. This data confirmed our previous results obtained using an ELISA-PRA test for posttransplant monitoring [7].

The dynamics of anti-HLA Abs after transplantation, in patients with pre-Tx DSA, was rather decisive for the development of ABMR. Patients, in whom DSA-MFI decreased/disappeared during the first two weeks, did not develop ABMR. Those who sustained their levels presented 22% of ABMR. On the other hand, 85% of patients, in whom the MFIs increased early after transplantation, developed ABMR.

Interestingly, in the ABMR group, we observed an iDSA-MFI sharp drop on the fourth day, a feature also shown by others [13], and then an increase between the 7th and the 14th day posttransplant. This finding suggests that collecting DSA before 4 days after transplant does not add important information. Our data suggests that DSA should be monitored at days 7 and 14 for sensitized patients. To our knowledge this profile had never been well described before.

For sensitized patients, we suggest monitoring PRA frequently (2-3 times) in the first month, beginning between the 7th and the 10th POD, when there is a greater variability on the behavior of the anti-HLA Abs, and then periodically over the first year.

In DSA positive patients, no significant (higher than 20%) change in the iDSA-MFI could be detected after the 2nd postoperative month, all of them evolving with progressive and slow decrease and no rejection episodes. It is noteworthy that the kinetics of these antibodies is very dynamic and can fluctuate lightly at any time, as shown by Heilman et al. [14], but as we have shown a pattern is usually defined during the first month.

DSA measure is a noninvasive procedure and may help in the early diagnosis of ABMR, resulting in a strong impact on graft survival [1, 2, 7, 11, 15, 16]. In addition, we observed that monitoring DSA helped to follow patients treated for ABMR.

Our study has a caveat on establishing the exact ABMR rate, since protocol biopsies were not performed in all low risk patients. Also, we used MFI-DSA as a marker of humoral activity, since this has been used in many published studies and is more useful in the clinical setting. Nevertheless, dilution studies could add more accurate data on the strength of the antibodies.

We could identify some risk factors for the early increase in DSA-MFI: female gender, previous pregnancies, retransplants, blood transfusions, and receiving a transplant from a deceased donor were all risk factors for increasing posttransplant DSAs.

In other studies, DSA presented at both pre-and posttransplant periods was a significant risk factor for decreased graft survival [11].

We noticed that patients without antibody-mediated rejection had better graft function, at one year, than those with ABMR, although one-year graft survival was similar.

Considering that renal allograft function is a surrogate marker for long-term graft loss [17], this finding may suggest that one-year follow-up is a too short period to evaluate the impact of ABMR on graft survival.

Since 70% of sensitized patients and 64% of patients with positive pretransplant DSA did not present ABMR and have good graft survival and function at least during the first year, sensitized patients should not be excluded for transplantation even if they show pretransplant DSA.

In summary, knowing the dynamics of anti-HLA Abs after transplantation in sensitized patients may allow us to recognize those at higher risk for rejection, ABMR, as early as possible.

## Figures and Tables

**Table 1:** Baseline patient characteristics.

PRE-TX groups (*N* = 150)	Group A	Group B	Group C	*p*
	89 (59.3%)	39 (26%)	22 (14.7%)	
Recipient age (y)	45.1 (±13.5)	50.5 (±13)	44 (±10)	**0.066**
Donor age (y)	42.7 (±11.5)	44.1 (±10.5)	46.7 (±11.3)	**0.313**
Dialysis pre-TX: yes	84 (94.4%)	36 (92.3%)	22 (100%)	**0.521**
Gender: male	64 (71.9%)	14 (35.1%)	5 (22.7%)	**<0.001**
Donor: deceased	54 (60.7%)	30 (76.9%)	15 (68.2%)	**0.198**
First transplant	86 (96.6%)	36 (92.3%)	13 (59.1%)	<0.001
Pregnancy: Yes (at least 1)	14 (56.0%)	23 (92.0%)	14 (82.4%)	<0.001
Transfusion: Yes (at least 1)	36 (40.4%)	22 (56.4%)	14 (63.6%)	**0.068**
Mean PRA class I	0	6%	35%	<0.01
Mean PRA class II	0	12%	34%	<0.01
Induction: thymoglobulin	11 (12.4%)	12 (30.8%)	19 (86.4%)	**<0.001**

PRA: panel reactive antibody.

**Table 2:** Clinical outcomes according to pretransplant PRA.

	Group A	Group B	Group C	*p*
	PRA = 0	PRA > 0 no DSA	PRA > 0 with DSA	
	*N* (89) = 59.3%	*N* (39) = 26%	*N* (22) = 14.7%	
Acute rejection	12/89 (13.5%)	10/39 (25.6%)	8/22 (36.4%)	<0.001
(i) ABMR	0 (0%)	4 (40%)	8 (100%)	<0.001
(ii) TCMR	12 (100%)	6 (60%)	0 (0%)	<0.001
Graft Loss	1/12 (8.3%)	1/10 (10%)	1/8 (12.5%)	0.999
Death	0/12 (0%)	0/10 (0%)	1/8 (12.5%)	-* *-* *-* *-

PRA: panel reactive antibody; DSA: donor specific antibody; ABMR: antibody-mediated rejection; TCMR: T-cell mediated rejection.

## Abbreviations

ABMR: Antibody-mediated rejection
Abs: Antibodies
AGH: Anti-human globulin
CDC: Complement dependent cytotoxicity
CDC-AHG: Complement dependent cytotoxicity-antihuman-globulin enhanced
DSA: Donor-specific antibodies
DTT: Dithiothreitol
ELISA: Enzyme-Linked Immune Sorbent Assay
FCXM: Flow-cytometry cross-match
HLA: Human leukocyte antigens
i-DSA: Immunodominant DSA
IVIg: Intravenous polyvalent immunoglobulin
KTx: Kidney transplantation
MFI: Mean fluorescence intensity
MM: Mismatch
POD: Postoperative day
PCR-SSO: Polymerase chain reaction sequence specific oligonucleotides
PCR- SSP: Polymerase Chain reaction single strand polymorphisms
PRA: Panel reactive antibodies
TCMR: T-cell mediated rejection
XM: Cross-match.

## References

[1] Terasaki P. I., Ozawa M. (2004). Predicting kidney graft failure by HLA antibodies: a prospective trial. *American Journal of Transplantation*.

[2] Lefaucheur C., Suberbielle-Boissel C., Hill G. S. (2008). Clinical relevance of preformed HLA donor-specific antibodies in kidney transplantation. *American Journal of Transplantation*.

[3] Colvin R. B. (2007). Antibody-mediated renal allograft rejection: Diagnosis and pathogenesis. *Journal of the American Society of Nephrology*.

[4] Tsapepas D. S., Vasilescu R., Tanriover B. (2014). Preformed donor-specific antibodies and risk of antibody-mediated rejection in repeat renal transplantation. *Transplantation*.

[5] Williams R. C., Opelz G., McGarvey C. J., Weil E. J., Chakkera H. A. (2016). The risk of transplant failure with HLA mismatch in first adult kidney allografts from deceased donors. *Transplantation*.

[6] Loupy A., Hill G. S., Jordan S. C. (2012). The impact of donor-specific anti-HLA antibodies on late kidney allograft failure. *Nature Reviews Nephrology*.

[7] de Souza P. S., David-Neto E., Panajotopolous N. (2014). Dynamics of anti-human leukocyte antigen antibodies after renal transplantation and their impact on graft outcome. *Clinical Transplantation*.

[8] David-Neto E., Souza P. S., Panajotopoulos N. (2012). The impact of pretransplant donor-specific antibodies on graft outcome in renal transplantation: A six-year follow-up study. *Clinics*.

[9] Sis B., Mengel M., Haas M. (2010). Banff '09 meeting report: antibody mediated graft deterioration and implementation of Banff working groups. *American Journal of Transplantation*.

[10] Tait B. D., Süsal C., Gebel H. M. (2013). Consensus guidelines on the testing and clinical management issues associated with HLA and non-HLA antibodies in transplantation. *Transplantation*.

[11] Loupy A., Jordan S. C. (2013). Transplantation: donor-specific HLA antibodies and renal allograft failure. *Nature Reviews Nephrology*.

[12] Loupy A., Lefaucheur C., Vernerey D. (2013). Complement-binding anti-HLA antibodies and kidney-allograft survival. *The New England Journal of Medicine*.

[13] Burns J. M., Cornell L. D., Perry D. K. (2008). Alloantibody levels and acute humoral rejection early after positive crossmatch kidney transplantation. *American Journal of Transplantation*.

[14] Heilman R. L., Nijim A., Desmarteau Y. M. (2014). De novo donor-specific human leukocyte antigen antibodies early after kidney transplantation. *Transplantation*.

[15] Campos É. F., Tedesco-Silva H., Machado P. G., Franco M., Medina-Pestana J. O., Gerbase-DeLima M. (2006). Post-transplant anti-HLA class II antibodies as risk factor for late kidney allograft failure. *American Journal of Transplantation*.

[16] Eng H. S., Bennett G., Bardy P., Coghlan P., Russ G. R., Coates P. T. H. (2009). Clinical significance of anti-HLA antibodies detected by Luminex®: Enhancing the interpretation of CDC-BXM and important post-transplantation monitoring tools. *Human Immunology*.

[17] Hariharan S., McBride M. A., Cherikh W. S., Tolleris C. B., Bresnahan B. A., Johnson C. P. (2002). Post-transplant renal function in the first year predicts long-term kidney transplant survival. *Kidney International*.

